# Role of Diet as a Predisposing Factor for Dilated Cardiomyopathy in Dogs: A Narrative Review

**DOI:** 10.3390/vetsci12111106

**Published:** 2025-11-20

**Authors:** Léa Mornard, Anna Carolina Massara Brasileiro, Mário Marcondes-Santos

**Affiliations:** 1 Department of Veterinary Sciences, EUVG—Vasco da Gama University School, Avenida José R. Sousa Fernandes 197, 3020-210 Coimbra, Portugal; anna.brasileiro@gmail.com; 2 CIVG—Vasco da Gama Research Center, Avenida José R. Sousa Fernandes 197, 3020-210 Coimbra, Portugal; 3 School of Agriculture, Santarém Polytechnic University, Quinta do Galinheiro—S. Pedro, 2001-904 Santarém, Portugal

**Keywords:** cardiac function, dietary fiber, dilated cardiomyopathy, dog, grain-free diet, gut microbiota, legumes, non-traditional diet, taurine

## Abstract

In 2018, the Food and Drug Administration (FDA) reported cases of dilated cardiomyopathy (DCM) in dogs, including breeds without known genetic predisposition, fed diets containing a high proportion of legumes or potatoes, many labeled grain-free. Despite concerns, grain-free diets remain increasingly popular. This narrative review focuses on the peer-reviewed literature and summarizes recent studies evaluating the effects of grain-free diets on canine cardiac health, with emphasis on taurine deficiency, metabolic alterations, and emerging hypotheses extending beyond nutritional inadequacy. These findings suggest a strong link between diet and DCM, particularly regarding legumes. Dogs of various breeds showed larger left ventricular diameters, reduced systolic function, and increased premature ventricular complexes when fed non-traditional, grain-free, legume-rich diets compared to those on traditional, low-legume diets. Many affected dogs improved clinically and functionally with dietary changes and treatment, suggesting nutritional DCM could be reversible. Though mechanisms remain unclear, the research highlights the potential roles of legumes, particularly peas, in the gut microbiota and fiber-related bile acid metabolism. This review also distinguishes taurine-deficiency-related DCM as a separate form, with certain breeds, notably Golden Retrievers, being more susceptible. Overall, further studies are required to better understand the role of nutrition in canine cardiac health.

## Simple Summary

In recent years, an increasing number of dogs have developed dilated cardiomyopathy while eating certain grain-free diets rich in legumes like peas and lentils. The aim of this article is to review and discuss recent scientific research to understand whether these diets could be causing this disease in dogs and what mechanisms could be involved. These findings showed that many apparently healthy dogs eating grain-free diets have early- stage heart modifications, indicating a possible causal effect. Various studies also showed that many affected dogs improved after switching to more traditional diets and receiving treatment, suggesting that this form of the condition may be reversible. The research also points to the possible role of ingredients such as peas and fiber and their effects on gut bacteria and nutrient absorption. This article highlights the impact of diet on the development of heart disease in dogs and supports the need for further research. This narrative review aims to help pet owners, veterinarians, and pet food companies make better decisions to protect the cardiovascular health of dogs.

## 1. Introduction

Dilated cardiomyopathy (DCM) is the second most common acquired cardiac disease in dogs, following myxomatous mitral valve degeneration [1]. It affects approximately 0.5% of the canine population, predominantly large-breed dogs such as the *Doberman pinscher*, as well as some medium-sized breeds, including the *Cocker spaniel* [2]. While DCM has traditionally been regarded as a predominantly hereditary disorder, it can also develop secondary to other factors, including nutritional deficiencies, endocrine disease, myocarditis, and chronic tachycardia. Increasingly, however, evidence suggests that nutrition may play a critical role in both the onset and progression of disease.

In recent years, veterinary cardiologists have reported a growing number of DCM cases in breeds with no known genetic predisposition. The issue gained public attention in 2018 when the FDA, in response to cardiologists’ concerns, issued a public notice and launched an investigation into the matter. Current understanding indicates, in addition to non-diet-related cases, DCM may arise in two diet-associated forms: one linked to taurine deficiency, and another related to yet unidentified dietary factors [3]. In response to this increase in cases, several studies have sought to investigate the association between diet and DCM. These studies have predominantly focused on grain-free/high pulse diets and their common ingredients, such as legumes (e.g., peas and lentils) [3,4,5,6,7,8,9,10,11]. Additional investigations have examined the role of taurine metabolism and potential breed predispositions to taurine deficiency [12,13]. More recent hypotheses suggest the gut microbiota as a contributing factor to cardiac health [14,15,16].

This article aims to provide a comprehensive narrative literature review of recent published studies that describe the association between diet and DCM in dogs. The main objective is to discuss the findings on grain-free diets’ impact on cardiac health, emerging theories beyond nutritional deficiency, and the clinical and dietary implications that may advance our understanding of nutrition’s role in canine heart disease.

## 2. Dilated Cardiomyopathy in Dogs

### 2.1. Definition

Dilated cardiomyopathy (DCM) is a condition characterized by thinning of the ventricular walls and dilation of the ventricular chambers, which may progress to generalized cardiomegaly. These morphological changes lead to reduced contractility during systole, volume overload, and low cardiac output, ultimately resulting in congestive heart failure (CHF) [17]. DCM is often divided into two main phases: an occult phase and a symptomatic phase. Although there is no universally accepted classification system for DCM in dogs, some authors have proposed an adaptation of the human CHF classification developed by the New York Heart Association (1994) [18]. In this system, stage I corresponds to no limitation of physical activity; stage II indicates slight limitation; stage III indicates marked limitation, although the patient remains comfortable at rest; and stage IV is characterized by the presence of CHF symptoms even at rest, with any physical effort exacerbating discomfort. Wess (2022) [19] proposed a canine-specific staging system for DCM, with stages A, B1, B2, C, and D.

### 2.2. Clinical and Histopathological Manifestations

DCM progresses through several stages, beginning with a prolonged asymptomatic period known as the occult phase. This stage is characterized by morphological changes and electrical abnormalities such as premature ventricular contractions (PVCs) [19].

Overall, the clinical presentation of DCM is highly heterogeneous, depending on age of onset, disease progression rate, and genetic and environmental factors, all of which contribute to the specific phenotypic expression [20]. However, the phenotype tends to be more homogeneous within individual breeds. According to the staging proposed by Wess (2022) [19], stage A is characterized by the absence of morphological and electrical abnormalities. Stage B refers to the occult phase and is subdivided into B1 and B2. In stage B1, dogs show no increase in cardiac size but in some cases, it is possible to present electrical abnormalities such as PVCs. Stage B2 dogs remain asymptomatic but display left ventricular systolic dysfunction, with or without increased left ventricular diastolic volume. Stage C includes patients with current or previous clinical signs of CHF, while stage D represents the terminal phase, characterized by signs refractory to standard treatment.

Histopathological examination of cardiac tissue in DCM reveals two main patterns: the attenuated wavy fiber type and the fatty infiltration type [21].

### 2.3. Primary and Secondary DCM

Several causes are associated with DCM, the most frequently diagnosed being the hereditary or primary form, which accounts for approximately 90% of reported DCM cases [22]. It affects specific breeds, such as the *Doberman pinscher*, as well as the *Irish wolfhound*, *Boxer*, *Portuguese water dog*, *Great dane*, and *Newfoundland* [22].

Secondary DCM results from an underlying condition, disease, or external factor that progressively impairs myocardial function, rather than a direct primary genetic defect. Reported causes include infectious, inflammatory, and endocrine diseases; arrhythmias; nutritional deficiencies; and exposure to toxins, heavy metals, or cardiotoxic drugs [23].

### 2.4. Diagnosis and Treatment

Echocardiography is essential for distinguishing between stages B1 and B2 and for guiding treatment. In stage B2, DCM is diagnosed based on reduced systolic function and secondary volume overload [19]. Systolic dysfunction is indicated by an increased left ventricular end-systolic volume (ESV), increased E-point-to-septal separation (EPSS), and reduced ejection fraction (EF), with EF < 40% indicating systolic impairment. A left ventricular fractional shortening (FS) below 20–25% is also indicative of systolic dysfunction. A sphericity index (SI) < 1.65 is considered abnormal. In stage B1, dogs may exhibit arrhythmias without the characteristic changes detectable by echocardiography that occur in stage B2. Therefore, 24 h Holter monitoring, which enables tracking of PVCs, can allow for the early detection of DCM at this stage. In general, >100 PCVs in 24 h was recommended as the cut-off value for establishing a diagnosis of DCM [19].

Blood-based biomarkers, including the N-terminal fragment of B-type natriuretic peptide (NT-proBNP), which predicts early cardiac enlargement, and cardiac troponin I (cTnI), a specific marker of myocardial injury, serve as useful screening tools for DCM. However, they do not replace Holter monitoring or echocardiography [19].

The primary goals of DCM treatment are to prevent progression to the symptomatic phase and to control cardiomyopathy and CHF once clinical signs are present. Early treatment is particularly important in predisposed breeds, involving the use of supraventricular antiarrhythmics such as digoxin, diltiazem, or amiodarone, and sotalol for ventricular arrhythmias [22]. In *Doberman pinschers*, pimobendan, an inotropic agent with vasodilatory properties, has demonstrated efficacy in treating asymptomatic cases [22]. In clinical stages, therapy aims to reduce preload and afterload while maintaining systemic blood pressure. Vasodilators, such as angiotensin-converting enzyme inhibitors (ACEIs), and inotropes, such as pimobendan, are often combined. Diuretics are indicated to alleviate congestion and reduce volume overload [22]. In secondary DCM cases, treatment should be tailored to address the underlying cause.

## 3. Diet and Cardiac Health in Dogs

In July 2018, the U.S. Food and Drug Administration (FDA) announced an investigation into cases of DCM in dogs associated with certain diets, many of which were marketed as “grain-free” [24]. These products frequently contained high levels of peas, lentils, legume seeds, and/or potatoes, which were often among the first listed ingredients. Notably, many affected dogs were of breeds without a known genetic predisposition to DCM, and in some cases, two dogs from the same household were affected [24].

### Non-Traditional Diets

A non-traditional diet refers to any feeding regimen that deviates from typical commercial formulations designed to meet the nutritional guidelines established by the European Pet Food Industry Federation (FEDIAF) or the Association of American Feed Control Officials (AAFCO). Such diets include grain-free, raw, home-prepared, and vegetarian or vegan food, as well as those produced by smaller companies that may incorporate unconventional ingredients such as exotic meats (e.g., *kangaroo*, *bison*). Among these, grain-free diets have emerged as the dominant trend in recent years. These formulations exclude common grains such as wheat, corn, rice, or barley, replacing them with alternative carbohydrate sources such as peas, lentils, or potatoes.

The growing popularity of grain-free diets appears to stem from several factors. Owners often project their own dietary preferences, such as gluten-free or grain-free trends, onto their dogs. Additionally, heightened concerns regarding food allergies have led some owners to believe, incorrectly, that cereals are a common cause of allergies in dogs. However, studies demonstrate that most canine food allergies are associated with animal proteins, such as chicken or beef, rather than grains [25]. Marketing also plays a significant role, as grain-free products are frequently promoted as healthier alternatives, with grains portrayed as “fillers” or “by-products.” However, there is currently no scientific evidence supporting claims that they are harmful to the health of dogs.

Between 2012 and 2016, sales of grain-free dog food in the United States increased by 221% [25]. A study by O’Brien et al. (2024) [26] reported that 40.3% of dogs in the United States were fed grain-free diets, highlighting their widespread use.

Since July 2018, the FDA has published two updates on its investigation, showing an exponentially increased number of reported cases, and several studies describing dogs with suspected diet-related DCM have been published. In June 2019, the FDA reported that 560 dogs had been reported with possible cases of diet-related DCM. To better understand the diets associated with DCM cases, the FDA analyzed product labels, checking whether they were grain-free and whether they contained peas, other legumes, or potatoes (including sweet potatoes). The data revealed that more than 91% of the products identified were grain-free, 93% contained peas and/or lentils, and 42% contained potatoes. The sources of animal protein in the reported diets were highly diverse, with no predominant protein source identified [27]. In its most recent update, the FDA revealed that, as of November 2022, it had received 1382 reports of diet-related DCM cases [28].

## 4. Grain-Free vs. Grain-Inclusive Diets: Evidence from Clinical and Echocardiographic Studies

### 4.1. Echocardiographic and Electrocardiographic Findings

Echocardiographic and electrocardiographic evaluation plays a central role in diagnosing diet-associated DCM. Several studies have compared dogs fed grain-free (GF)/non-traditional diets (NTD) versus grain-inclusive (GI)/traditional diets (TD), while others have assessed outcomes following a dietary transition in dogs previously fed GF formulations [4,8,29,30]. Across these investigations, parameters such as fractional shortening (FS), ejection fraction (EF), left ventricular internal diameters in systole (LVIDs) and diastole (LVIDd), left atrial-to-aortic ratio (LA/Ao), E-point-to-septal separation (EPSS), and premature ventricular contractions (PVCs) were analyzed. Importantly, most affected dogs received conventional heart failure therapy—including pimobendan, ACE inhibitors, and furosemide—alongside dietary modification and, in some cases, taurine supplementation. The results of these studies are grouped into echocardiographic studies comparing healthy dogs fed TDs and NTDs, as illustrated in Table 1 [9,13], echocardiographic studies comparing dogs with DCM fed GF/NTDs and GI/TDs after dietary change, as shown in Table 2 [4,8,11,29], and echocardiographic and electrocardiographic studies comparing healthy or asymptomatic dogs fed GF and GI diets, as seen in Table 3.

Among the studies that compared dogs fed different diets, some findings are particularly relevant. For example, in the study by Ontiveros et al. (2020) [13], 86 *Golden Retrievers* were analyzed, divided into two groups: dogs fed traditional diets (TD; *n* = 43), which were grain-inclusive, not including legumes or potatoes in the top five ingredients, and those fed non-traditional diets (NTD; *n* = 43), which were typically grain-free and contained legumes. The researchers evaluated taurine concentrations and echocardiographic parameters in these dogs. The results indicated that the NTD group showed a reduction in EF and FS, a significant increase in LVIDs and LVIDd, and a higher end-systolic volume index (ESVI) compared to the GI group, indicating impaired ventricular function in these dogs. Similarly, Owens et al. (2023) [9] conducted a study comparing echocardiographic measurements and cardiac biomarkers (NT-proBNP, cTnI, whole blood taurine, plasma taurine) in 46 healthy dogs, including 23 fed TDs (grain-containing diets without potatoes or pulse ingredients among the top ten listed ingredients) and 23 fed NTDs (diets with pulses primary ingredients). The results showed that dogs in the NTD group had a lower mean EF and a higher left ventricular ESVI compared to dogs in the TD group.

Other studies, with research focused on the progression of echocardiographic measures after dietary change, have provided important data on the relationship between diet and DCM. For example, in the retrospective study by Adin et al. (2019) [4], dogs diagnosed with DCM over a three-year period were divided into those fed grain-free (GF; *n* = 36) and grain-inclusive (GI; *n* = 12) diets. The GF group was further subdivided into dogs consuming the most common GF diet, GF-1 (*n* = 14), and other GF diets, GF-0 (*n* = 22); the brand of these diets was not disclosed in the article. Seven dogs from the GF group were re-evaluated: five had their diet changed to a GI diet and two were switched to another GF diet from a major brand. All seven re-evaluated dogs showed clinical and echocardiographic improvement after the diet change, with reductions in LVIDdN, LVIDsN, and LA/Ao ratio. An important finding in this study was the identification of two pairs of unrelated cohabiting dogs, both consuming GF-1 that developed DCM, supporting diet as a common environmental factor in the development of DCM in these cases.

Another retrospective study, conducted by Freid et al. (2021) [29], analyzed medical records of dogs diagnosed with DCM over four years, classifying them into those fed GI (*n* = 15) and GF (*n* = 56) diets. Dogs in the GF group showed a higher frequency of congestive heart failure (CHF). Compared with GF dogs that did not change diet (*n* = 25; 18 with CHF) and the GI group, GF dogs that transitioned to GI diets (*n* = 31; 25 with CHF) exhibited a significant reduction in LVIDsN and LA/Ao ratio, suggesting an improvement in cardiac remodeling. Similarly, the retrospective study by Walker et al. (2021) [11] evaluated the association between diet and clinical progression in 67 dogs with DCM and CHF, divided into GF (*n* = 43) and GI (*n* = 24) groups. All dogs in the GF group had been consuming these diets for at least six months prior to diagnosis and underwent diet change as part of treatment. Unlike other studies, no significant differences in LVIDsN, EF, or LA/Ao ratio were observed between groups. However, dogs in the GF group demonstrated a significant reduction in LVIDdN after diet change. In addition, this group showed a greater reduction in EPSS, suggesting improved left ventricular systolic function.

The prospective study of Freeman et al. (2022) [8] evaluated echocardiographic changes in dogs with DCM fed NTDs (which were grain-free or included pulses or potatoes/sweet potatoes in the top ten ingredients) and TDs (which were grain-inclusive and had no pulses or potatoes/sweet potatoes in the top ten ingredients), as well as in dogs with subclinical cardiac changes (SCC) consuming NTDs. Among the 60 dogs with DCM and 16 dogs with SCC, most in the DCM group received medical treatment, and five in the SCC group received ≥1 cardiac medication (pimobendan [*n* = 3], carvedilol [*n* = 2], sotalol [*n* = 1]). Most dogs in both groups received taurine supplementation and diet change. Fifty-seven percent of NTD-fed dogs and 33% of TD-fed dogs survived until the nine-month follow-up. While the TD group showed no significant echocardiographic changes, NTD-fed dogs demonstrated an improvement in EF as well as reductions in LVIDdN, LVIDsN, and LA/Ao ratio, indicating recovery of cardiac function and remodeling. Similarly, dogs with SCC also showed improvements in the same parameters after nine months.

Another relevant study, conducted by Haimovitz et al. (2022) [30], investigated the effect of diet change in asymptomatic dogs with elevated cardiac biomarkers (hs-cTnI, NT-proBNP) or echocardiographic abnormalities. The study included 20 dogs, 10 GF-fed and 10 GI-fed. All were switched to a GI diet that excluded legumes and potatoes among the top 25 listed ingredients. Over one year, GF-fed dogs showed a progressive reduction in LVIDsN, while GI-fed dogs had no significant changes. In addition, one GF-fed dog with frequent PVCs at baseline showed a gradual reduction in frequency, from frequent at baseline to intermittent at the third assessment and absent in subsequent evaluations. This finding is particularly interesting, as it suggests a possible reversal of PVCs following dietary modification.

These findings align with those of Adin et al. (2021) [5], which analyzed the effects of different diets in four dog breeds: two without known genetic predisposition to DCM (*Miniature schnauzer* and *Whippet*), one with recognized hereditary DCM (*Doberman pinscher*), and one breed considered at increased risk of nutritional DCM (*Golden retriever*). Echocardiograms, cardiac biomarkers (NT-proBNP, hs-cTnI) and blood/plasma taurine concentrations were compared between GF-fed (*n* = 26) and GI-fed (*n* = 162) dogs. In addition, diets were categorized according to the presence of FDA-listed ingredients of concern (peas, lentils, or potatoes) among the top ten listed ingredients (FDA-PLP; *n* = 39) versus those without such ingredients (NoFDA-PLP; *n* = 149). Results showed no echocardiographic differences between healthy dogs fed GF versus GI diets, nor between FDA-PLP and NoFDA-PLP diets. However, a higher prevalence of PVCs was observed in dogs consuming FDA-PLP diets (10%) compared with NoFDA-PLP diets (2%). This finding is supported by a recent study on the subject, by Coppinger et al. (2024) [7], which retrospectively analyzed echocardiographic and electrocardiographic findings in apparently healthy Irish Wolfhounds. Ninety-seven dogs were compared, 35 (36%) fed legume-rich diets and 62 (64%) fed legume-poor diets. No significant differences were found between groups regarding prevalence of atrial fibrillation or echocardiographic measures. However, a significantly higher percentage of dogs in the legume-rich group (17%) had PVCs compared with the legume-poor group (2%).

Although not all studies found the same echocardiographic or electrocardiographic changes, this variability may be explained by differences in sample size, duration of GF diet exposure prior to study enrollment, and intervals between re-evaluations. Nevertheless, most studies point to consistent trends: GF-fed dogs tend to have higher LVIDdN and LVIDsN, lower fractional shortening, lower EF, and higher ESVI compared with GI-fed dogs. A particularly interesting finding is that these cardiac changes appear to be partially reversible. Several studies demonstrated that after switching to a GI diet, some dogs showed improvement in echocardiographic parameters, including reductions in LVIDdN, LVIDsN, LA/Ao ratio, and EPSS, and improvements in EF [4,8,11,30]. Another important aspect of these studies is that GF-fed dogs appear to have an increased risk of developing PVCs. This finding is significant, as PVCs may represent one of the earliest signs of structural heart disease, reinforcing the importance of cardiac evaluation using some diagnostic tests (such as electrocardiogram, Holter monitoring, echocardiogram, and cardiac biomarkers) in dogs fed grain-free diets.

### 4.2. Taurine and Amino Acid Levels

In most studies, taurine supplementation was incorporated into treatment regardless of the patients’ baseline levels. Although the majority of studies assessing taurine deficiency did not identify a significant number of deficient dogs, some research reports present noteworthy findings.

The study developed by Freid et al. (2021) [29], which demonstrated that GF-fed dogs who underwent dietary change had a significant reduction in LVIDsN and LA/Ao ratio, also showed that dogs receiving taurine supplementation had a greater reduction in LVIDsN, but not in the LA/Ao ratio, compared with dogs that did not receive supplementation.

In the study carried out by Adin et al. (2019) [4], 61% of GF-fed dogs and 42% of GI-fed dogs had whole-blood taurine and L-carnitine concentrations tested. None of the GF-fed dogs were deficient. Of the seven dogs reevaluated after dietary change, six received taurine supplementation and showed clinical and echocardiographic improvement three months after diagnosis. The only dog that did not receive taurine supplementation showed minimal echocardiographic improvement during this same period but presented more evident improvement nine months after diagnosis. This result suggests that recovery is possible, albeit slower, without supplementation. The authors of this study also highlighted that several GF-fed dogs had elevated whole-blood taurine concentrations prior to any supplementation. Such elevated values are unexpected in these cases and new studies should be conducted to investigate this finding. One hypothesis is that the high taurine levels observed in these dogs may be associated with myocardial injury, as after myocardial cell death, cardiomyocytes release taurine, which is partially taken up by circulating platelets. In the study conducted by Adin et al. (2021) [5], GF-fed dogs had higher plasma taurine levels compared with GI-fed dogs; however, whole-blood taurine concentrations were not elevated. Circulating taurine levels have been used as a biomarker of myocardial injury in humans, although whole-blood taurine concentrations appear to be more closely associated with injury than plasma concentrations. These results, however, remain unexplained [5]. One explanation proposed by the authors is that this could be due to the inclusion of taurine or methionine in diets, or alternatively, could be related to myocardial injury.

Considering these findings, it remains uncertain to what extent the echocardiographic improvement observed in these studies was due to taurine supplementation or to dietary change, since most of these dogs’ diets were supplemented. It is possible that, even in the presence of normal taurine levels, supplementation provided a survival benefit or contributed to the improvement of echocardiographic parameters. However, in the study of Freeman et al. (2022) [8], no dogs presented with reduced plasma or whole-blood taurine concentrations. On the contrary, many dogs with DCM in both the NTD and TD groups, as well as dogs in the SCC group, had elevated plasma or whole-blood taurine levels. Taurine supplementation was initiated at the initial evaluation for most dogs but later discontinued if laboratory results did not indicate a need. The main finding of this study was that improvement in EF over time was significantly associated with prior consumption of an NTD, with no differences between groups in the proportion of dogs receiving taurine supplementation or in the duration of supplementation. Thus, the positive effects observed on EF appear to be more related to dietary change (to a grain-inclusive traditional diet) than to taurine supplementation.

These various studies showed that taurine deficiency is rare in both GF- and GI-fed dogs. However, contrary to what might be expected, some dogs present with elevated taurine levels, which have not yet been fully explained. A possible justification is that these elevated levels may originate from myocardial injury. Freeman et al. (2022) [8] demonstrated that echocardiographic parameter improvement can occur solely due to dietary change. However, Adin et al. (2019) [4] suggested that taurine supplementation may accelerate this recovery process.

### 4.3. Troponin I, NT-ProBNP

Several studies have analyzed the cardiac biomarkers cTnI and NT-proBNP to assess whether there is a correlation between their values and taurine levels, and echocardiographic findings (such as an increase in ESVI, LVIDs, LVIDd, and LA/Ao ratio or a reduction in EPSS, EF, SI, and FS). Although few studies have investigated NT-proBNP, none identified significant differences between GF-fed and GI-fed dogs over time. This may be due to the fact that many animals included in these studies were at early stages of the disease, without severe remodeling. Conversely, cTnI has been more frequently investigated and has yielded more relevant results, as described below.

The study conducted by Owens et al. (2023) [9] did not find increases or significant differences between groups regarding NT-proBNP or cTnI concentrations. In contrast, Walker et al. (2022) [11] evaluated cTnI concentrations in eleven dogs with DCM, five fed GF diets and six fed GI diets. All dogs had elevated cTnI concentrations above the established reference interval (0.2 ng/mL).

Adin et al. (2021) [5] reported that healthy GF-fed dogs showed higher median plasma taurine concentrations compared with GI-fed dogs and also exhibited significantly higher hs-cTnI levels. This finding was replicated when comparing FDA-PLP and NoFDA-PLP diet groups, reinforcing the hypothesis that GF diets may be associated with a greater degree of subclinical myocardial injury. Although no echocardiographic differences were identified between groups, one hypothesis proposed was that high plasma taurine levels in these dogs could be a consequence of cardiomyocyte injury, supported by the concomitant increase in hs-cTnI observed in these animals, potentially indicating an early process of cardiac damage.

These findings were further supported by Haimovitz et al. (2022) [30], who observed a progressive reduction in hs-cTnI concentrations in GF-fed dogs following transition to a conventional diet. In contrast, GI-fed dogs showed either stable hs-cTnI concentrations or only minimal increases over time. The median hs-cTnI values (ng/mL) in GF-fed dogs were 0.141 (interquartile range [IQR]: 0.012–0.224) at baseline and 0.092 (IQR: 0.044–0.137) after one year, whereas in GI-fed dogs they were 0.051 (IQR: 0.016–0.195) at baseline and 0.060 (IQR: 0.022–0.280) after one year. The reduction in hs-cTnI concentrations following dietary change strengthens the hypothesis of a nutritional influence in DCM and suggests that the initial elevations observed in GF-fed dogs were abnormal and may carry clinical relevance.

The study carried out by Freeman et al. (2022) [8], which also identified elevated taurine concentrations in several dogs, corroborates these findings by demonstrating a significant reduction in hs-cTnI concentrations between baseline and nine months after dietary change in the NTD group. In the TD group, no significant changes were observed over the same period. Furthermore, this study found a weak correlation between taurine and hs-cTnI concentrations, suggesting, as noted by Adin et al. (2021) [5], that elevated taurine levels may be related to myocardial damage.

### 4.4. Reversibility of Diet-Associated DCM

The analysis of clinical and echocardiographic parameters in dogs with diet-associated DCM following the implementation of a treatment plan allows for am assessment of the potential benefits of dietary modification and taurine supplementation, as well as providing evidence for possible disease reversibility in these cases.

Freid et al. (2021) [29] demonstrated that GF-fed dogs that underwent a dietary change exhibited a significantly greater reduction in LVIDsN and LA/Ao ratio compared with those that remained on the same diet, reinforcing the positive impact of dietary modification.

Similarly, Adin et al. (2019) [4] observed that GF-fed dogs had higher LVIDdN and LVIDsN values compared with the GI group. None of the GF-fed dogs required an increased dose of diuretics or experienced recurrence of CHF after the dietary change. Furthermore, three dogs had furosemide discontinued between six and nine months after dietary modification. These results indicate that diet-associated DCM may occur with certain GF diets and that dietary modification can contribute to improvement.

In the study of Walker et al. (2022) [11], outcomes were worse in GF-fed dogs that had been on such diets for longer periods prior to diagnosis or were diagnosed at a younger age. However, these dogs showed significantly greater improvement in LVIDdN and EPSS, as well as a significant reduction in furosemide and pimobendan dosages over time, compared with GI-fed dogs. These findings indicate reversal of ventricular remodeling in the GF group compared with the GI group, suggesting that nutritional DCM associated with GF diets may be reversible.

Haimovitz et al. (2022) [30] further supported this hypothesis by demonstrating reductions in both hs-cTnI and LVIDsN levels in GF-fed dogs after dietary modification, providing evidence for reversibility of subclinical myocardial changes. Similar results were reported by Freeman et al. (2022) [8], who found that dogs with DCM or SCC previously fed NTDs showed mild but significant improvements in echocardiographic parameters after dietary change during the nine-month study period. Improvement in EF over time was significantly associated with prior NTD consumption. However, these dogs continued to exhibit a high risk of sudden death, similar to that seen in dogs with primary DCM. These findings are consistent across studies, suggesting that in GF/NTD -fed dogs with DCM, dietary modification may be associated with significant improvements in some echocardiographic measures.

The reduction in hs-cTnI levels, along with clinical and echocardiographic improvement and increased survival following dietary change, provides strong evidence that partially reversible DCM may occur in dogs fed GF diets. This finding contrasts with GI-fed dogs, in which the absence of improvement suggests primary DCM. The reversal of cardiac remodeling appears to be related not only to appropriate management of DCM but also, and primarily, to dietary modification. Nevertheless, the role of taurine supplementation—even in the presence of normal blood taurine concentrations—is not yet fully understood, although it may provide additional therapeutic benefit. It is noteworthy that some of the breeds most predisposed to primary DCM, such as *Doberman pinschers*, *Great danes*, and *Boxers*, were included in these studies. Still, in some cases, echocardiographic improvements were observed in predisposed breeds, reinforcing the likely hypothesis that diet may influence disease development even in dogs genetically predisposed to primary DCM.

## 5. Breed-Specific Considerations on Diet and DCM

A predisposition to taurine deficiency has been identified in several breeds, including *Golden retrievers*, *Newfoundlands*, *Cocker spaniels*, *English setters*, and *Labrador retrievers* [12]. This increased risk in certain breeds suggests possible genetic or metabolic differences that render them more vulnerable to taurine depletion. In recent years, *Golden retrievers* have been disproportionately represented among cases of diet-associated DCM reported to the FDA, prompting more in-depth investigation.

Kaplan et al. (2018) [12] examined the clinical, dietary, and echocardiographic characteristics of *Golden retrievers* with taurine deficiency and DCM to identify specific dietary associations. The authors also assessed whole-blood taurine concentrations in healthy dogs. Among 24 dogs diagnosed with taurine deficiency and DCM, 23 were fed GF diets rich in legumes, none of which had been tested under AAFCO feeding protocols. After dietary modification and taurine supplementation, 23 dogs showed echocardiographic improvement and normalization of taurine concentrations. Of the 24 affected dogs, 11 were diagnosed with CHF, nine of whom showed resolution of congestion upon reevaluation. In this subgroup, diuretic therapy was discontinued in five dogs and reduced by more than 50% in four others. In the control group of 52 apparently healthy *Golden retrievers*, a high prevalence of low whole-blood taurine was observed. This suggests that taurine deficiency, and potentially DCM, may be more common than currently estimated and that the breed may have specific susceptibility to the condition. Because echocardiography was not performed in these control dogs, it was not possible to determine whether any had subclinical DCM.

These findings were reinforced by a subsequent study by Ontiveros et al. (2020) [13], who evaluated the risk of *Golden retrievers*, fed non-traditional grain-free diets high in legumes or potatoes, developing taurine deficiency and nutritional DCM, compared with those consuming traditional commercial diets. The authors also proposed breed-specific reference intervals for whole-blood and plasma taurine concentrations. The study assessed 43 dogs in each group and found that NT-fed *Golden retrievers* had significantly lower whole-blood taurine concentrations, although plasma levels did not differ between groups. In addition, NT-fed dogs exhibited lower EF, higher LVIDd and LVIDs, and increased ESV compared with the TD group. Reference intervals for taurine were established using the TD group, all of which had normal echocardiograms. For whole blood, the reference interval was 213–377 nmol/mL (90% CI: lower limit 198–230; upper limit 355–396), and for plasma, 63–194 nmol/mL. These findings suggest that *Golden retrievers* may physiologically have higher taurine concentrations than those reported in previous multi-breed studies, indicating that taurine deficiency in this breed may have been underdiagnosed. Although the sample size for defining reference intervals was moderate (*n* = 43), larger studies are warranted to validate these results and further investigate the causal mechanisms. Importantly, the study also highlighted that NT-fed *Golden retrievers* exhibited significantly reduced taurine levels and a higher frequency of systolic dysfunction compared with TD-fed dogs.

Although taurine is not currently considered an essential amino acid for dogs, its deficiency has long been linked to DCM, with certain breeds being more predisposed to the condition. The studies reviewed demonstrate that grain-free diets containing legumes increase the risk of taurine deficiency and cardiac abnormalities in *Golden retrievers*. The study conducted by Ontiveros et al. (2020) [13] further suggests that reference intervals for taurine concentrations may differ across breeds, indicating that a single universal reference range cannot be applied to all. The increased risk of deficiency is likely multifactorial, involving a combination of dietary, metabolic, and genetic factors.

## 6. New Theories Beyond Nutritional Deficiency

### 6.1. Food Analysis

Despite ongoing research efforts, the influence of diet on observed cases of DCM remains not fully understood. Traditional nutritional analyses of implicated diets have failed to identify a causal factor, indicating the need for more innovative approaches. One emerging method for studying the relationship between diet and disease is metabolic analysis. Although metabolites are typically assessed in plasma or urine, this method can also be applied to the evaluation of biochemical compounds in foods themselves, a field referred to as “*foodomics*” [31].

In their study, Smith et al. (2021) [31] aimed to identify biochemical compounds that differ between commercial dog foods associated with DCM and more traditional commercial diets. They conducted a metabolic profile analysis of nine diets associated with canine DCM (containing ≥ three legumes, potatoes, or sweet potatoes as main ingredients) belonging to the 16 most frequently reported brands linked to DCM cases submitted to the FDA (the 3P/FDA group). The comparison group consisted of nine non-3P/FDA diets. A total of 830 biochemical compounds (665 of known identity and 165 of unknown identity) were quantified and compared between the two diet groups. Of these, 88 compounds were present at higher concentrations and 23 at lower concentrations in the 3P/FDA group. The largest categories of differing compounds were amino acid-related (*n* = 24), amino acid derivatives, and xenobiotics/plant-derived compounds (*n* = 20). Three unnamed compounds (X-23541, X-23534, and X-25605) were detected exclusively in the 3P/FDA diet group. Random Forest analysis identified the top 30 compounds distinguishing the two diet groups, all of which were elevated in the 3P/FDA group. Taurine concentrations were not significantly different between groups.

Among the ingredients that distinguished the two groups, peas, lentils, chicken/turkey, and rice were most notable. Peas were the ingredient most strongly associated with many of the compounds elevated in the 3P/FDA diets. Potatoes and sweet potatoes were not sufficiently represented in either group to allow for meaningful evaluation.

One possible hypothesis regarding how 3P/FDA diets may contribute to DCM is that they could be insufficient in essential nutrients required for maintaining cardiovascular health. With respect to nutrient insufficiency, several compounds relevant to cardiac metabolism, including B vitamins, were lower in the 3P/FDA diets. B vitamins act as cofactors in numerous reactions essential for cardiac metabolism. For example, vitamins B6 and B12 are cofactors in carnitine and taurine synthesis; thus, a deficiency or insufficiency in these vitamins could potentially contribute to DCM.

This study represents a significant advancement, being the first to apply an innovative *foodomics* approach to analyze diets associated with DCM. It provides valuable information about ingredients and biochemical compounds that may adversely affect canine cardiovascular health.

### 6.2. Metabolic Profile and Dietary Compounds

One key question raised by the *foodomics* analysis is whether the biochemical compounds that distinguish the two types of diets are also detectable in dogs consuming diets associated or not with DCM. If metabolic analysis identifies the same elevated compounds both in DCM-associated diets and in the circulation of dogs with DCM fed these diets, this could suggest a potential causal role of these compounds in the disease.

To investigate this, Adin et al. (2022) [6] conducted a study comparing metabolic profiles of dogs fed GF vs. GI diets. At baseline, differences in lipid and amino acid metabolism were observed between the GF and GI groups, with 46 metabolites higher and 82 lower in the GF group. After 12 months of dietary change, these differences decreased significantly, leaving only six metabolites elevated and 11 reduced metabolites in the GF group. The results showed similarities to the previous *foodomics* study, including lower levels of vitamins and cofactors and higher levels of xenobiotics in DCM-associated diets. The study also identified several yet-unnamed compounds that may serve as biomarkers of GF diet consumption, though their clinical significance remains unknown. Among these, metabolite X-25419 was particularly notable, being 6.65-fold higher in the GF group compared with the GI group. This same compound had already been identified in Smith et al. (2021) [31], with a 7.67-fold increase, reinforcing that diet is the likely source of this metabolite in circulation. Although it is not surprising that dietary compounds are detectable in the blood of dogs, the metabolic profile of dogs is not expected to mirror that of the food due to changes associated with absorption and metabolism. This study demonstrates that consumption of GF diets alters the biochemical profile of dogs.

Another study, conducted by Smith et al. (2022) [32], investigated metabolic differences in dogs with DCM. The analysis included dogs with DCM, 38 consuming NTDs (grain-free or included pulses or potatoes in the top 10 ingredients) and eight consuming TDs (grain-inclusive and had no pulses or potatoes in the top 10 ingredients), and healthy controls, 12 consuming NTDs and 17 consuming TDs. In addition, metabolic changes were evaluated after nine months of dietary intervention and medical treatment. A total of 153 metabolic differences were identified between dogs with DCM and healthy controls, and 63 differences between dogs fed NTD and TD. Twelve metabolites overlapped in both analyses, suggesting that NTDs may affect metabolic pathways relevant to cardiac disease. Among the 17 metabolites most altered in dogs with DCM consuming NTD, many were involved in fatty acid and amino acid metabolism, oxidative stress, and inflammation, indicating possible distinct mechanisms for primary versus nutritional DCM. Most of these metabolites decreased following dietary change, accompanied by clinical and echocardiographic improvement. X-25419 again stood out, being significantly elevated in dogs with DCM consuming NTD, but decreasing after dietary change.

In addition, carnitine derivatives were elevated in the blood of dogs with DCM, suggesting that carnitine transport or metabolism may be impaired. Protein–protein interaction networks and functional enrichment analysis of gene networks identified 105 significant metabolic pathways, including those related to aging, inflammation (nuclear factor kappa B, tumor necrosis factor alpha, and interleukin-18), and oxidative stress, supporting the hypothesis that inflammatory processes play a relevant role in nutritional DCM.

Based on these findings, the authors proposed that nutritional DCM may result more from an excess of certain dietary compounds than from a simple nutrient deficiency. This hypothesis could explain the gradual recovery observed in these dogs, in contrast with simple deficiencies, which typically respond rapidly to supplementation. One possible mechanism is that high levels of specific dietary compounds interfere with the bioavailability of essential cardiac nutrients such as carnitine. Although carnitine itself did not differ between diets in previous studies, metabolites elevated in NTD—such as D-carnitine, acetyl-D, L-carnitine, deoxycarnitine, choline, and betaine—may inhibit its absorption via the SLC22A5 transporter, thereby reducing its availability to the myocardium.

These metabolic assessments highlight significant biochemical differences between GF and GI diets, as well as between dogs consuming them, particularly given that these differences diminish after dietary change. Metabolites elevated both in DCM-associated diets and in the blood of affected dogs may represent potential causal factors. Moreover, the recurrent presence of unidentified metabolites, such as X-25419, underscores the need for further research to determine their identity and clinical relevance.

### 6.3. Bile Acid Metabolism and Microbiota Alterations

Compared with cereals, legumes contain relatively high concentrations of soluble fiber (including oligosaccharides), which can bind to bile acids in the intestine, increasing their excretion. Oligosaccharides also serve as a direct energy source for microbiota, promoting microbial proliferation. This process may enhance taurine loss due to microbial metabolism, as bile acids are primarily conjugated with taurine in the liver before being secreted into the intestine as taurocholic acid. Once in the intestine, microbial actions on bile acids (such as deconjugation) can affect taurine [14]. When bile acids are deconjugated, taurine is released, a portion may be reabsorbed into the bloodstream, while a portion may be lost in the feces. Recent research suggests that dietary components such as soluble and insoluble fiber, bile acids, and the intestinal microbiota may contribute to nutritional DCM in dogs.

Pezzali et al. (2020) [16] compared GI and GF diets in Beagles and found that GI diets led to greater excretion of primary bile acids (25.49% for GF vs. 12.09% for GI on day 28 of the study). These findings suggest that the higher soluble fiber content, including oligosaccharides, in GF diets may modify the fecal bile acid pool, likely through alterations in gut microbiota composition.

Similarly, Clark et al. (2023) [15] reported that a low-animal-protein GF diet induced significant changes in fecal microbiota, an increase in short-chain fatty acids and primary bile acids, and a reduction in secondary bile acids.

Quilliam et al. (2023) [10] compared three diets formulated with white rice flour (GI), lentils (GF), or peas (GF). They found that the pea-based diet, characterized by high levels of oligosaccharides, amylopectin, and high-molecular-weight insoluble dietary fiber (IHMWDF), impaired cardiac function (increased LVIDs and decreased cardiac output) and reduced macronutrient digestibility. This diet also resulted in the lowest total fecal bile acid excretion, suggesting a potential relationship between dietary fiber, bile acid metabolism, and early-stage DCM. Notably, these changes were not observed in the lentil-based diet. These findings align with the *foodomics* study, in which peas were more frequently associated with problematic diets than lentils, pointing toward a possible link between dietary amylopectin, oligosaccharides, IHMWDF, and nutritional DCM in dogs.

Bokshowan et al. (2023) [14] tested the effects of oligosaccharides on cardiac biomarkers and found that although dietary raffinose (the primary oligosaccharide found in peas) increased NT-proBNP levels, it did not immediately impair cardiac function. However, higher NT-proBNP levels, reduced ejection fraction, and increased LVIDs were observed in dogs fed a dental diet rich in insoluble fiber, which decreased total bile acid excretion. This supports the concept that IHMWDF may limit bile acid reabsorption and impact taurine metabolism.

Although the exact mechanisms remain unclear, these studies suggest that IHMWDF and oligosaccharides may contribute to DCM by influencing bile acid metabolism, intestinal microbiota balance, and potentially taurine availability. Further research is needed to determine how these interactions evolve over time

## 7. Survival Time and Long-Term Outcomes

Median survival time for dogs diagnosed with DCM varies depending on factors such as breed, disease severity at diagnosis, and treatment protocols. In general, DCM carries a poor long-term prognosis. A study by Martin, Johnson, and Celona (2009) [33] reported that, among 354 dogs for which survival analysis was possible, median survival time was 19 weeks (IQR: 4–60 weeks), with survival rates of 28% one year after diagnosis and 14% after two years.

In cases of nutritional DCM, recent studies suggest that diet change can significantly improve survival outcomes. Freid et al. (2021) [29] reported a median survival time of 337 days (range: 9–1307 days) for dogs consuming GF diets who underwent dietary change, compared with 215 days (range: 1–852 days) for those that remained on the original diet. Similarly, Freeman et al. (2022) [8] found that dogs diagnosed with DCM had a median survival time of 611 days (range: 2–940 days) in the NTD group, compared with only 161 days (range: 12–669 days) in the TD group. Walker et al. (2022) [11] further supported these findings, reporting median survival times of 344 days (interquartile range: 88–666 days) for dogs previously fed GF diets versus 253 days (interquartile range: 70–380 days) for those on GI diets.

In addition, several studies reported that dogs previously fed GF diets were able to reduce dosages of cardiac medications over time, and some even discontinued diuretics and pimobendan after dietary modification [4,11,12]. These results suggest that dogs consuming GF diets who subsequently develop DCM may have a better prognosis, longer survival times, and reduced medication requirements after a diet change compared with dogs with DCM already consuming GI diets.

## 8. Conclusions

Current evidence supports an association between grain-free diets and the development of DCM in dogs. Several studies indicate that these diet-associated cardiac changes can improve—sometimes substantially—after dietary modification to a grain-inclusive formulation, particularly when combined with appropriate medical management. Such findings emphasize that nutritional DCM may represent a potentially reversible form of the disease when identified early.

The link between grain-free diets and cardiac remodeling highlights the importance of detailed dietary history and early diagnostic assessment, including echocardiography and cardiac biomarkers, in dogs presenting with compatible clinical signs. Early recognition allows for timely dietary intervention, which may improve ventricular function and overall prognosis.

Some breeds—such as Golden Retrievers—have a genetic predisposition to taurine-deficient DCM. This breed is also more vulnerable to nutritional DCM, as those fed grain-free diets show a higher risk of taurine deficiency.

In summary, diet plays a critical role in the pathogenesis and potential reversibility of some forms of canine DCM. Continued research is needed to clarify the metabolic and nutritional mechanisms involved, refine diagnostic strategies, and guide evidence-based dietary recommendations aimed at protecting the cardiovascular health of dogs

## Figures and Tables

**Table 1:** Results of echocardiographic studies comparing healthy dogs fed NTDs and TDs.

Study	Number of Dogs	Compared Diets	Main Changes Observed in the NTD Group
Ontiveros et al. (2020) [13]	86	NTD (*n* = 43) vs. TD (*n* = 43)	↓ FS, ↓ EF, ↑ LVIDs and LVIDd, ↑ ESVI
Owens et al. (2023) [9]	46	NTD (*n* = 23) vs. TD (*n* = 23)	↓ EF, ↑ ESVI

EF, ejection fraction; ESVI, end-systolic volume index; FS, fractional shortening; LVIDd, left ventricular internal diameter in diastole; LVIDs, left ventricular internal diameter in systole; NTD, non-traditional diet; TD, traditional diet.

**Table 2:** Results of echocardiographic studies comparing dogs with DCM fed GF diets/NTDs and GI diets/TDs after dietary change.

Study	Number of Dogs	Compared Diets	Main Changes Observed in the GF/NTD Group
Freid et al. (2021) [29]	71	GF (*n* = 56) vs. GI (*n* = 15)	↓ LVIDsN and LA/Ao ratio
Adin et al. (2019) [4]	48	GF (*n* = 36) vs. GI (*n* = 12)	↓ LVIDdN and LVIDsN, LA/Ao ratio
Walker et al. (2021) [11]	67	GF (*n* = 43) vs. GI (*n* = 24)	↓ LVIDdN and ↓ EPSS
Freeman et al. (2022) [8]	20	NTD (*n* = 51) vs. TD (*n* = 9)	↑ FS, ↓ LVIDdN and LVIDsN, ↓ LA/Ao ratio

EPSS, E-point to septal separation; FS, fractional shortening; GF, grain-free diet; GI, grain-inclusive diet; LA/Ao ratio, left atrium-to-aorta ratio; LVIDdN, left ventricular internal diameter in diastole normalized to bodyweight; LVIDsN, left ventricular internal diameter in systole normalized to bodyweight; NTD, non-traditional diet; TD, traditional diet.

**Table 3:** Results of echocardiographic and electrocardiographic studies comparing healthy or asymptomatic dogs fed GF diets/diets rich in legumes and GI diets/diet low in legumes.

Study	Number of Dogs	Compared Diets	Main Changes Observed in the GF Group
Haimovitz et al. (2022) [30]	71	GF (*n* = 10) vs. GI (*n* = 10)	After diet change ↓ LVIDsN, ↓ PVCs in one dog
Adin et al. (2021) [5]	48	GF (*n* = 26) vs. GI (*n* = 162) and FDA-PLP (*n* = 39) vs. NoFDA-PLP (*n* = 149)	PVCs: FDA-PLP 10% vs. NoFDA-PLP 2%
Coppinger et al. (2024) [7]	67	Diet rich in legumes (*n* = 35) vs. diet low in legumes (*n* = 62)	PVCs: 17% vs. 2%

FDA-PLP, diets containing FDA-listed ingredients of concern (peas, lentils, or potatoes) among the top 10 listed ingredients; GF, grain-free diet; GI, grain-inclusive diet; LVIDdN, left ventricular internal diameter in diastole normalized to bodyweight; NoFDA-PLP, diets without FDA-listed ingredients of concern (peas, lentils, or potatoes) among the top 10 listed ingredients; PVCs, premature ventricular contractions.

## Data Availability

No new data were created or analyzed in this study. Data sharing is not applicable to this article.

## Abbreviations

The following abbreviations are used in this manuscript:

AAFCO	Association of American Feed Control Officials
ACEI	Angiotensin-converting enzyme inhibitors
ACS	Subclinical cardiac alterations
cTnI	Cardiac troponin I
CHF	Congestive heart failure
CI	Confidence interval
DCM	Dilated cardiomyopathy
EF	Ejection fraction
EPSS	E-point to septal separation
ESVI	End-systolic volume index
FDA	U.S. Food and Drug Administration
FDA-PLP	Diets containing FDA-listed ingredients of concern (peas, lentils, or potatoes) among the top 10 listed ingredient
FEDIAF	European Pet Food Industry Federation (from French *Fédération Européenne de l’Industrie des aliments pour Animaux Familiers*)
FS	Fractional shortening
GF	Grain-free diets
GI	Grain-inclusive diets
Hs-cTnI	High-sensitivity cardiac troponin I
IHMWDF	Insoluble high-molecular-weight dietary fiber
IQR	Interquartile range
LA/Ao	Left atrium-to-aorta ratio
LVIDd	Left ventricular internal diameter in diastole
LVIDdN	Left ventricular internal diameter in diastole normalized to bodyweight
LVIDs	Left ventricular internal diameter in systole
LVIDsN	Left ventricular internal diameter in systole normalized to bodyweight
NoFDA-PLP	Diets without FDA-listed ingredients of concern (peas, lentils, or potatoes) among the top 10 listed ingredients
NTD	Non-traditional diets
NT-proBNP	N-terminal pro-B-type natriuretic peptide
SI	Sphericity index
TD	Traditional diets
PVCs	Premature ventricular contractions
3P/FDA	Diets containing ≥ three legumes, potatoes, or sweet potatoes as main ingredients, within the 16 major dog food brands most frequently associated with reported cases of canine DCM to the FDA
